# *In Vivo*, Evaluation of Wound Healing Activity of Nanoliposomes Loaded *Withania somnifera* Extract

**DOI:** 10.34172/apb.42403

**Published:** 2024-12-05

**Authors:** Mohadese Mirshekari, Azar Bagheri Ghomi, Hamed Hamishehkar, Mohammad Reza Farahpour

**Affiliations:** ^1^Department of Chemistry, Central Tehran Branch, Islamic Azad University, Tehran, Iran.; ^2^Drug Applied Research Center, Tabriz University of Medical Sciences, Tabriz, Iran.; ^3^Research Center of New Material and Green Chemistry, Khazar University, 41 Mehseti Street, AZ1096, Baku, Azerbaijan.; ^4^Department of Clinical Sciences, Faculty of Veterinary Medicine, Urmia Branch, Islamic Azad University, Urmia, Iran.

**Keywords:** Angiogenesis, Antibacterial, Antioxidant, Epithelialization, Nanoparticle, Wound healing

## Abstract

**Purpose:**

Medicinal plants and their derivatives have been used to treat wounds, and loading the plants into nanoliposomes (NLPs) helps to increase their efficacy. This study investigated the efficacy of NLPs loaded with *Withania somnifera* (WHSE) extract in mouse models for excisional wound healing.

**Methods:**

In the present study, we thoroughly evaluated WHSE’s antibacterial, antioxidant, and safety profiles. Additionally, we assessed wound contraction, pathological evaluations, and the expression of basic fibroblast growth factor (bFGF) and CD31.

**Results:**

The results showed that the extract and its NLPs had biocompatibility and exhibited antibacterial and antioxidant properties. Furthermore, our in vivo wound healing assay results showed that ointments containing 0.50% and 1.00% of the WHSE-NLPs accelerated wound healing and increased collagen and epithelialization. Furthermore, the results of the immunofluorescence and immunochemical tests indicated more expression of CD31 and bFGF in the mice that have been treated with WHSE-NLPs compared to those who were treated with WHSE and control groups. (*P*<0.05).

**Conclusion:**

We demonstrated that the administration of 1.00% of the WHSE-NLPs could compete with the commercial ointment (Nitrofurazone®). Therefore, balms prepared from WHSE-NLPs expedited the wound healing process by increasing collagen, epithelialization, and the expression of CD31 and bFGF.

## Introduction

 The skin, the body’s largest organ, serves as the primary barrier against harmful pathogens. Physical or chemical damage to the skin can disrupt this biological barrier, leading to severe injuries like dehydration, protein loss, and bacterial infections.^1^ Wound healing involves a complex process, including homeostasis, inflammation, proliferation, and remodeling.^2^ However, the natural wound healing process can be time-consuming due to bacterial infection, and the administration of antibiotics can lead to antibiotic resistance.^3^ Biological agents, such as plant compounds, have been used for centuries to treat wounds. These compounds have antibacterial, proangiogenic, wound-stretching, and anti-inflammatory properties, making them valuable tools in healing.^4,5^

 WHSE, commonly known as Ashwagandha, is a multipurpose medicinal plant belonging to the Solanaceae family. It is abundantly found in subtropical regions around the world. Folk healers have traditionally used this plant to treat various ailments, including fever, cancer, asthma, diabetes, stomach ulcers, hepatitis, eye ulcers, arthritis, heart problems, and hemorrhoids. Ashwagandha is known for its anti-cancer properties and for treating back pain and toning muscles, which may be attributed to the alkaloids known as anolides. Furthermore, WHSE is rich in valuable secondary metabolites, including steroids, alkaloids, flavonoids, phenols, saponins, and glycosides. Various clinical trials have attributed different properties to Ashwagandha, such as heart protection, anticancer effects, antioxidant, antibacterial, antifungal, anti-inflammatory, liver protection, anti-depressant, blood sugar-lowering, antimicrobial, and wound-healing properties.^6-8^ Moreover, different parts of the plant have been evaluated for potential treatments for male infertility, obsessive-compulsive disorder, anti-anxiety effects, bone and muscle strengthening, blood lipid reduction, and anti-diabetes properties.^6^

 Active compounds found in WHSE include withaferin A, viscosalactone B, 27-deoxy withaferin A, pubesenolide, jaborosalactone D, 4b,27-dihydroxy-l-oxo-22R-witha-2,5,24-triptolide, 2,3-dihydro withaferin A-3b-O-sulfate, withanolide A, and 27-hydroxy withanolide B.^9^ Studies have reported on the wound-healing activity of WHSE.^8,10^ However, plant-derived agents have limitations, such as volatile structures and insolubility in water.^11^ The situation requires the creation of new agents that can surpass these constraints.

 Many natural products are unstable and have low oral bioavailability, which limits their potential for medicinal use.^12^ A nanotechnology-based approach has been proposed involving the incorporation of plant extracts into nanostructured drug delivery systems. These systems exhibit different physical, chemical, and biological properties at the nanoscale compared to the microscale, including enhanced optical properties and larger contact surfaces.^13^

 Liposomes are important lipid-based carriers that can hold various materials with different polarities. They are non-toxic, biodegradable, and don’t trigger the immune system. Liposomes also offer advantages such as high encapsulation efficiency, controlled and targeted release, simple manufacturing, and high stability. Nanoliposomes (NLPs) have a larger surface area than liposomes, resulting in better solubility and stability, increased bioavailability, and more precise delivery to target areas. Because of their amphipathic structure, the antioxidant agents contained in NLPs can prevent the onset of the oxidation process at the interface between water and oil in food.^14^

 The traditional use of medicinal plants faces several challenges. For instance, the oxidation of effective substances in the plant, its extract, and essential oil can have inappropriate effects on target and non-target tissues. However, there is hope in introducing drug-delivery nanocarriers such as NLPs. These recent strategies can significantly enhance the therapeutic effects of plants and their extracts.^15^

 Evidence suggests that crude plant extracts often exhibit better anti-plasmodial activity in vitro or in vivo than isolated compounds at equivalent doses. The study aimed to investigate the antibacterial properties and wound-healing activity of NLPs loaded with WHSE extract, which has potential anti-inflammatory properties. The study assessed the physicochemical and antioxidant properties, wound healing activity, and expression of specific proteins in an excisional wound model in mice.

## Materials and Methods

###  Materials

 The entire WHSE plant was harvested from the mountains in Saravan County, located in the Sistan and Baluchestan Province of Iran in September. We purchased the following materials from Sigma-Aldrich: soybean lecithin, cholesterol, ethanol (99.6%), xanthan gum, 4’,6-diamidino-2-phenylindole (DAPI), 2,2-diphenyl-1-picrylhydrazyl (DPPH), Folin–Ciocalteu reagent, sodium carbonate, gallic acid, rutin, aluminum chloride, sodium hydroxide, resazurin reagent, and nutrient broth medium. Additionally, all immunofluorescence and immunohistochemistry reagents were purchased from Santa Cruz Biotechnology, Inc. in Dallas, Texas.

###  Preparation of herbal extract

 The WHSE plant was harvested, and the Research Center of Medicinal and Ornamental Plants at Sistan and Baluchistan University confirmed its quality. The plant’s leaves were cut and washed with cold water to remove any dust. The washed leaves were then dried in a dark place at 37 °C. After drying, the leaves were ground into a powder using ball milling equipment. The prepared herbal powder was placed in the upper chamber of a Soxhlet extractor, and 400 mL of ethanol was added to the balloon. The extraction process was carried out for 9 hours. We used the rotary evaporator to vaporize the solvent, storing the residue at -20 °C.

###  FTIR analysis

 The Shimadzu Fourier transform infrared (FT-IR) spectrophotometer (Japan) was used to conduct FT-IR analysis on the powdered extract. The extract powder was mixed with an equal weight of KBr and compressed into tablets using a tablet preparation press. The sample was then placed into the FT-IR spectrophotometer at a temperature of 26 ± 1 °C, and infrared rays were scanned in the range of 400-4000 cm^-1^.^16^

###  Measurement of total flavonoid content

 To determine the total flavonoid content, we used the aluminum chloride colorimetric method.^17^ To prepare the mixture, we mixed 0.5 mL of the sample solution, 0.15 mL of 5% (w/v) aluminum chloride, and 0.15 mL of 10% (w/v) sodium hydroxide. Next, we added 3.2 mL of deionized water and 1 mL of 4% (w/v) NaOH to the mixture. The prepared mixture was incubated for 20 minutes, after which the absorbance of the samples was measured at 510 nm using a visible spectrophotometer (Pharmacia biotech-ULTRAS PEC2000-UK). The flavonoid content was measured using a calibration curve constructed with rutin as the standard flavonoid reagent. Finally, the flavonoid content was reported in milligrams of rutin equivalent per gram.

###  Measurement of total phenol content

 To determine the phenolic content, we used the Folin-Ciocalteu reagent. Firstly, 2.5 mg of Folin-Ciocalteu reagent (0.2 N) was added to 0.5 mL of a 10 mg/mL extract. After a five-minute incubation, 2 mL of a 75 g/L sodium carbonate solution was added to the mixture. The prepared mixture was then incubated for 2 hours, and its absorbance was measured at 750 nm using a UV-spectrophotometer. The phenolic content was quantified using a calibration curve constructed with gallic acid as the standard phenolic reagent. Afterward, the sample’s absorbance at 517 nm was measured using a spectrophotometer.^18^

###  Preparation for measuring antioxidant efficiency 

 The DPPH assay technique was used to determine the extract’s ability to scavenge free radicals. The procedure involved mixing equal volumes of different concentrations of the extract samples (50, 100, 200, 300, 400, 500, and 1000 µg/mL) with a DPPH solution (2 mg/50 mL). The mixture was then incubated in a dark place for 30 minutes. The absorbance of the samples was measured using a spectrophotometer at 517 nm.^19^

###  The preparation of liposomes by thin-layer hydration method

 To prepare liposomes, we used the thin-layer hydration method. Initially, 10 mg of extract was liquefied in 20 mL of ethanol. Then, lecithin (90 mg), cholesterol (10 mg), and Tween®80 (10 mg) were added to the extract solution and thoroughly mixed. The round-bottom flask was used to transfer the mixture of lipids and extracts, and the ethanol was removed by vacuum evaporation at 45 °C using a rotary evaporator. Once a thin layer formed at the bottom of the flask, the coating was hydrated using 10 mL of 1X PBS solution. Finally, for size reduction, the liposomes were subjected to homogenization using a Silent Crusher M homogenizer (Germany) for 20 minutes at 60 °C and 20,000 RPM.^20^

###  Drug loading and entrapment efficiency

 We used an ultrafiltration technique to separate the un-loaded extract and measured its concentration using spectrophotometry to determine the loading efficacy and capacity. Initially, standard samples of the extract with logarithmic concentrations were prepared, and then the UV uptake was measured at 412 nm for each sample. These absorbance values were used to calculate the slope of the line equation. Subsequently, the unloaded extract was separated using an Amicon® Ultra-4 100 k – 30 kDa molecular weight cut-off membrane (Millipore, Billerica, MA). We calculated the loading capacity and effectiveness using the following formula:


Eq. (1) $$\begin{array}{l} Loading\;capacity=\frac{loaded\;drug\;weight-Free\;amount\;of\;drug}{carrier\;weight}\times 100\\ :\;loading\;efficiency\;of\;liposomes \end{array}$$

###  Determination of the size and zeta potential of nanoparticles

 Dynamic light scattering (DLS) is a technique used to measure the size and zeta potential of NLPs. It utilizes the Brownian motion within a solution to measure the hydrodynamic diameter of the NLPs, providing information about their size in the solution. Scanning electron microscopy (SEM) and transmission electron microscopy (TEM) confirm the actual size of nanoparticles. Zeta potential displays the stability of NLPs based on their surface charge, playing a significant role in understanding interactions with the Malvern Instruments Ltd. DLS Zeta Sizer 2000 instrument was utilized to determine NLPs’ particle size distribution and peak size range.

###  Scanning electron microscope

 SEM was employed to determine the actual size of the prepared liposomes. To perform this analysis, a 1/100 diluted drop of the sample was placed onto an aluminum sheet and allowed to dry at room temperature. The dried samples were subsequently examined using an SEM instrument (TEscan, VEGA II XMU, Czech Republic).

###  Liposome stability

 In order to determine the stability of the liposomes containing the extract, the sample was refrigerated to prevent the destructive effects of temperature on the liposomes’ phenolic compounds. The sample was kept at 4 °C for 30 days and analyzed every 15 days. The stability of the liposomes was assessed through DLS tests conducted on the sample.^21^

###  Microbial analyses

 To assess the antibacterial effects of the extract and its NLPs, we determined the minimum bactericidal concentration (MBC) and minimum inhibitory concentration (MIC) against two types of bacteria, *Pseudomonas aeruginosa* (PTCC-1310) and *Staphylococcus aureus* (PTCC-1764), using the methods described in previous studies (Weinstein and Lewis, 2020). Serial dilutions of the crude extract and NLPs were prepared in a Nutrient broth medium. Subsequently, 100 μL of bacterial suspension was added to each tube at 37 °C for 24 hours. The growth inhibition of the bacteria was assessed by adding 30 µL of resazurin reagent to each well. The MIC refers to the lowest concentration of extract and its NLPs that can prevent the growth of microorganisms. On the other hand, MBC is the lowest extract concentration, and its NLPs can kill 99.9% of the bacterial population.

###  Animal study

 The surgical procedures and protocols utilized in this study were conducted by the guidelines established by the Ethical Committee of Islamic Azad University of Urmia University (No. 12041). 108 male mice weighing 25 ± 2 g were acquired for the in vivo experiment. The mice were individually housed in cages, maintained at 20-22 °C, and subjected to a 12-hour light-dark cycle.

 The mice were provided with standard rodent food and water at all times. Anesthesia was administered using appropriate anesthetic agents, ketamine (50 mg/kg), and xylazine (10 mg/kg); then, the back of each mouse was shaved. A 7 mm wound was then created in the back of each mouse using a circular full-thickness method, as described in previous studies.^22,23^ The study lasted 12 days, and treatments began 24 hours after the wounds were created.

 The mice were divided randomly into six different groups: (1) control group (Ctl) without any treatment, (2) Nitrofurazone (Ntfn) group as a positive control, (3) ointments containing NLPs, (4) WSHE, (5) 0.5% WSHE-NLPs, and (6) 1.00% WSHE-NLPs. Each group consisted of 18 mice.

###  Evaluation of wound healing

 The effectiveness of the treatments was assessed by measuring the reduction in wound size on days 3, 7, and 12 after the surgery. A camera was used to capture digital images of the wounds, which were then analyzed using an image analysis program by the methodology described in a prior study.^24^

###  Histological analysis of wound healing

 On days 3, 7, and 14, the animals were anesthetized using appropriate anesthetic agents, and skin wound samples were collected from the mice. Histological examinations were performed using the Trichrome Masson staining method, following established protocols described in previous studies.^25^ First, the collected samples were washed and fixed in a 10% formalin solution after being washed with saline and dehydrated. Then, the samples were processed by embedding them in clear xylene and wax and finally examined using light microscopy (BX50, Olympus, Japan).

 Based on previous studies, we conducted immunofluorescence staining using the described methods.^26^ Wound samples were collected, embedded in paraffin, dewaxed, and rehydrated. Antigen retrieval was performed by immersing the slides in citrate buffer (pH 7.4) for 20 minutes.The slides were first washed with Triton X-100 (0.03%) in TBS. Then, they were blocked with 10% normal serum or 1% BSA in TBS for 2 hours at room temperature. The slides were treated with primary antibody overnight at 4 °C. Then, the slides were washed three times with PBS (5 minutes each time) and incubated with a biotinylated secondary antibody for 1 hour at 37°C. After that, they were detected with 3,3-diaminobenzidine substrate. The ratio of target protein-positive nuclei to DAPI-positive nuclei in 3 microscopic fields per group was used for quantification using ImageJ software. Immunofluorescence staining was carried out to examine the expression of basic fibroblast growth factor (bFGF) and CD31 on days 7 and 14. The staining was done to evaluate the expression of bFGF and CD31 using the following antibodies: bFGF antibody (sc-365106, Santa Cruz Biotechnology, Inc), CD31 antibody (sc-365106, Santa Cruz Biotechnology, Inc), Goat Anti-Mouse IgG antibody (E-AB-1011, Elabscience Company), and Goat Anti-Rabbit IgG antibody (E-AB-1014, Elabscience Company).

###  Data analysis 

 The study data were analyzed for normality using the Kolmogorov-Smirnov test in SPSS software (version 23). As the data represented averages, a one-way ANOVA was performed. We used Duncan’s post hoc test to compare group differences with a *P* value of less than 0.05 for statistical significance.

## Results and Discussion

###  FTIR analysis of WHSE

**Figure 1:**
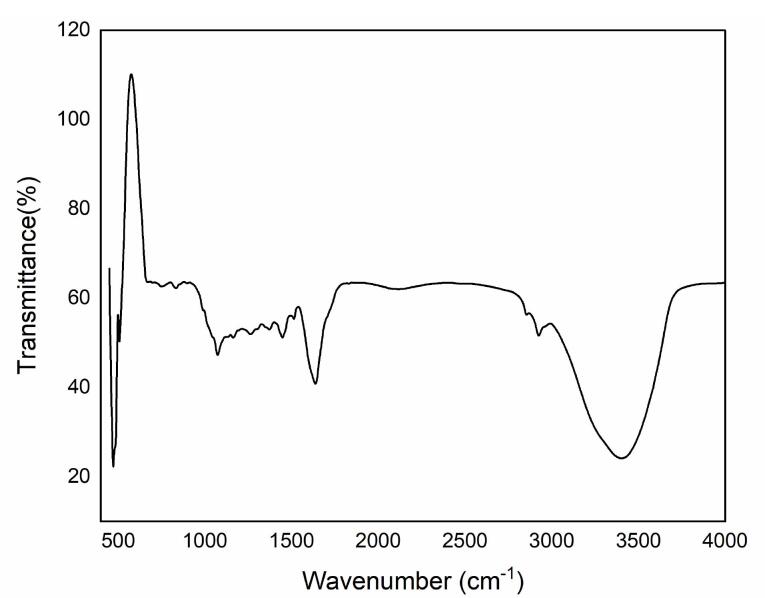
RepresentstheFT-IR scanning results of the WHSE extract

 FTIR analysis was conducted on the ethanolic extract of WHSE, which revealed distinct peaks in the FTIR spectrum (Figure 1). A peak at 3378.73 cm^-1^ indicated the presence of strong alcohol OH stretch bonds in the WHSE structure. Additionally, peaks at 2920.96 cm^-1^ and 2853.59 cm^-1^ were observed, representing a weak -C-H stretch characteristic of alkanes and alkyls present in the WHSE extract. Another peak at 1739.35 cm^-1^ indicated the presence of a potent C = O ester containing aldehyde. Moreover, a peak at 633.42 cm^-1^ suggested the presence of a strong C-Cl alkyl halide moiety. Notably, intense peaks at 1396.29 cm^-1^ and 1054.59 cm^-1^ indicated the presence of a strong CF alkyl halide moiety. Previous studies by Gupta et al in 2018 support these findings.^16^

###  Physicochemical characterization of WHSE loaded into the liposome

**Figure 2:**
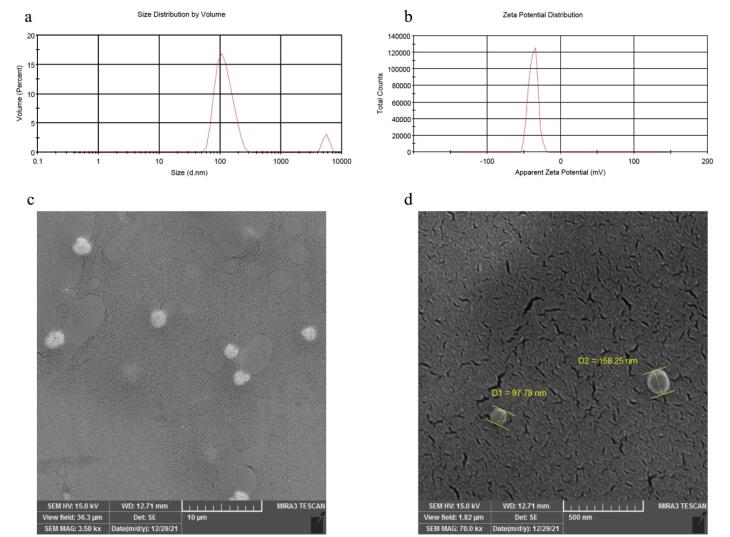
Represents (a) The size of the NLPs, (b) The zeta potential of NLPs, and (c and d) an SEM image of the morphology of NLPs in two different magnifications

 In Figure 2a, the actual size of WHSE-NLPs is depicted. Our findings demonstrate that the liposomes had an average diameter of approximately 120 nm and exhibited a spherical morphology. Furthermore, our SEM results were corroborated by DLS analysis, as shown in Figure 2b. The DLS measurements revealed that the mean hydrodynamic diameter of WHSE-loaded liposomes was 133 nm, accompanied by a narrow Polydispersity Index value of 0.3. Additionally, the zeta potential of the liposomes was determined to be -37.4 mV. These results collectively indicate the suitability of the NLPs for efficient drug delivery to the skin. Furthermore, our drug loading studies demonstrated a loading capacity of approximately 50% and an encapsulation efficiency of around 75%, which are considered satisfactory values for drug delivery systems.

 This study’s calculated encapsulation efficiency was higher than the reported data for nisin-loaded NLPs (54%).^27^ However, the study by Gibis et al showed that the encapsulation efficiency values for coated and uncoated NLPs containing grape seed extract were reported as 88% and 99%, respectively.^28^ Several factors influence the encapsulation efficiency of biologically active compounds in NLPs. These factors include the characteristics of the biologically active materials (such as lipophilicity or hydrophobicity and solubility), the properties of phospholipids (such as saturation and fatty acids), the ratio of phospholipids to the encapsulated compounds, the methods and conditions used in the production of NLPs, the concentration and type of stabilizers (like cholesterol), and environmental conditions such as temperature, pH, and ionic strength.^28^

 NLPs’ physicochemical characteristics and stability depend on factors such as pH, ionic strength, and temperature. The fluidity of the lipid membrane mainly influences the stability of NLPs. Due to their slight difference in density, NLPs neither settle nor float with the continuous phase; instead, Brownian motion keeps the liposomes in suspension. However, crowding and mixing are the primary factors contributing to instability and an increase in particle size. Consequently, one of the processes causing the instability of lipid systems is the accumulation of particles during storage. Adsorption forces between the particles cause them to stick together and form larger structures. If NLP particles undergo sedimentation and floating processes during the liposomal system maintenance, it indicates particles’ accumulation. At ambient temperature, due to the high fluidity of the membrane, liposomes agglomerate and form a clot, leading to an increase in particle size and a comprehensive and heterogeneous particle size distribution.

 According to the DLS results, a reading of 133 on the first day, 130 on the 15th day, and 137 on the 30th day shows that the liposomes remain stable and have a good shelf life when stored in the refrigerator. Changes in temperature and light can affect the lipid crystal structure, soften the membrane, and reduce the zeta potential, leading to particle aggregation due to decreased repulsive force between particles. Moreover, the materials composing the liposome membranes can impact the physical stability of the liposomes.

###  Antioxidant efficacy of extract using DPPH reagent

 Phenolic and flavonoid compounds have been shown to reduce lipid peroxidation, which leads to increased collagen fibril viability and enhanced strength of collagen fibers. As a result, cell damage is prevented and DNA synthesis is accelerated.^29^ Flavonoids, phenolics, and terpenoids are well-known promoters of the wound-healing process due to their antimicrobial and antioxidant properties. These properties contribute to wound contraction and accelerated epithelialization.^30^ Previous research has shown that the presence of reactive oxygen species hinders wound healing acceleration, while antioxidants neutralize free radicals and enhance the wound-healing process.^29,31^ Plant-derived antioxidants, such as polyphenolics, flavonoids, and tannins, act as potent scavengers of free radicals, thereby preventing oxidative damage during the wound-healing process.^32^

**Figure 3:**
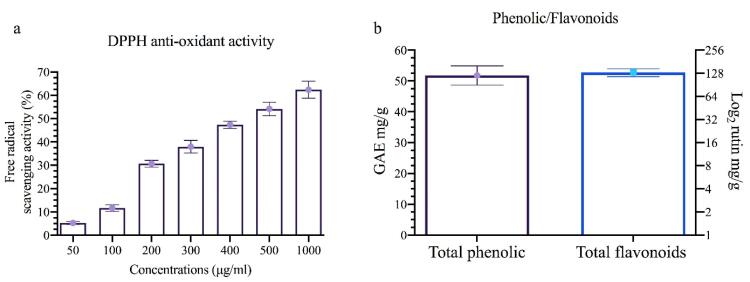
(a) Represents the antioxidant efficacy of extract using DPPH reagent, (b) the concentration of phenolic and flavonoids in each g of extract

 Our antioxidant activity measurements are depicted in Figure 3, revealing a concentration-dependent activity. Additionally, our measurements of total flavonoids and phenols indicated concentrations of approximately 51.80 GAE mg/g and 130.77 rutin mg/g in the WHSE extract. Previous studies on the antioxidant activity of WHSE are consistent with these results.^33,34^ The antioxidant properties of WHSE are due to the presence of phenolic and flavonoid compounds.In a study conducted by Ly et al,^35^ the antioxidant activity of Morinda citrifolia leaf extract was evaluated using the DPPH assay. Their results showed an IC50 concentration of 133.99 µg/mL DPPH. Furthermore, their study reported a flavonoid content of 2.649 mg RU g^−1^ of dry mass in the Noni leaf extract.

###  Antibacterial activities of the extract and its NLPs 

**Figure 4:**
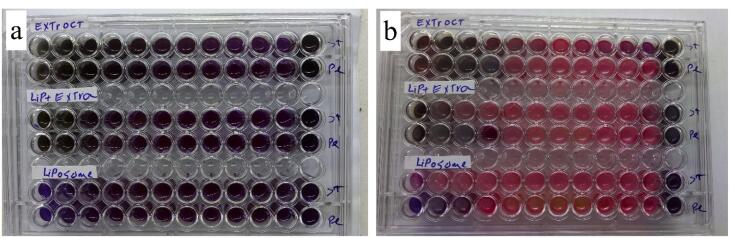
Antibacterial activity (a) MIC and (b) MBC of extract and its NLPs

 In Figure 4, the results indicate that WHSE, WHSE-NLP, and NLP exhibited bacteriostatic activity in the MIC test against both bacteria, starting from the lowest concentration of 0.156 µg/mL. The antibacterial activities of WHSE and WHSE-NLPs were observed against both bacteria in the range of 0.156-1.25 µg/mL. However, NLP only displayed antibacterial activity in the 0.625-0.156 µg/mL range against *P. aeruginosa*. These findings align with previous studies on the antibacterial activity of WHSE,^36,37^ which have attributed this activity to the active compounds present in WHSE. In a 2008 study, Gortzi et al developed liposomes encapsulated with Myrtus communis extract. The liposomes, prepared using chloroform and methanol, showed antioxidant and antimicrobial properties. The encapsulated extract demonstrated superior activity to the pure form of M. communis.^38^ Although it was expected that coating the extract with NLP would enhance the antibacterial activity, the study did not observe this result.

###  Wound healing activity 

 The process of wound healing is a complex mechanism that involves the restoration of cellular structures and tissue layers in damaged organs or tissues with the aim of achieving a normal physiological state. It starts with the fibroblastic stage, during which the wounded area begins to shrink.^39^ The wound-healing process typically consists of three stages: inflammation, proliferation, and remodeling. These stages are characterized by hemostasis and inflammation, cell proliferation and migration, epithelialization, angiogenesis, and collagen deposition, respectively. The size of the wound decreases during the remodeling stage.^40^ Various types of cells, including macrophages, keratinocytes, endothelial cells, and fibroblasts, are activated in the wound-healing mechanism. Successful wound closure requires cross-talk between healthy keratinocyte cells and other cells involved in wound healing.^41^ Plant metabolites with therapeutic properties can stimulate fibroblast and keratinocyte proliferation and growth, potentially accelerating the wound-healing process.^42^

**Figure 5:**
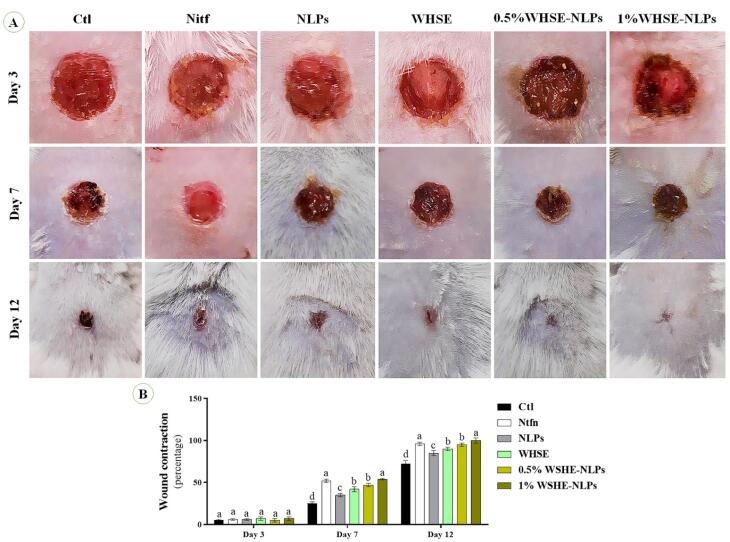
(A & B)Wound healing activity of the extract and its NLPs. Different superscripts (a-e) show significant differences on the same day. The data are presented as mean ± SD

Figure 5 illustrates the wound-healing activity. The results demonstrate that the treatment did not influence wound healing activity on day 3 (*P* = 0.615). However, on days 7 (*P* = 0.001) and 14 (*P* = 0.001), wound contraction was significantly higher in the Ntfn and 1% WSHE-NLPs groups compared to other groups. Wound contraction was also higher in the 0.5% WSHE-NLPs, WHSE, and NLPs groups compared to the control groups on days 7 (*P* = 0.001) and 14 (*P* = 0.001). Previous studies have reported similar findings regarding the wound-healing activity of WHSE.^8,10^ The results indicate that the administration of 1.00% WSHE-NLPs could be comparable to the commercial ointment Ntfn in promoting wound healing. Coating the extract with NLP increased wound healing activity compared to using uncoated WHSE. This improvement in wound healing activity can be attributed to the extract’s antibacterial action and antioxidant properties. Previous studies have reported the role of antioxidants and antibacterial compounds in accelerating the wound-healing process.^23,24^ Furthermore, the extract and its NLP formulation increased the expression of CD31 and collagen, which further facilitated the wound-healing process. Higher concentrations of the extract were found to improve wound healing compared to lower concentrations, likely due to a higher concentration of active compounds. In a study, the wound-healing activity of Morinda citrifolia leaf extract was evaluated. Their findings showed that the ethanolic extract of Morinda citrifolia could increase wound healing speed by 92.45% compared to the control group. The results also demonstrated that the extract had wound-healing effects in mice after 11 days of treatment by reducing the wound area and promoting histological regeneration.^35^

 The study conducted by Juneja et al examined the effects of Boerhavia diffusa extract on wound healing. Both the methanolic and chloroform extracts were found to increase human keratinocyte cell proliferation, both in vivo and in vitro.^42^

###  Pathological results 

**Figure 6:**
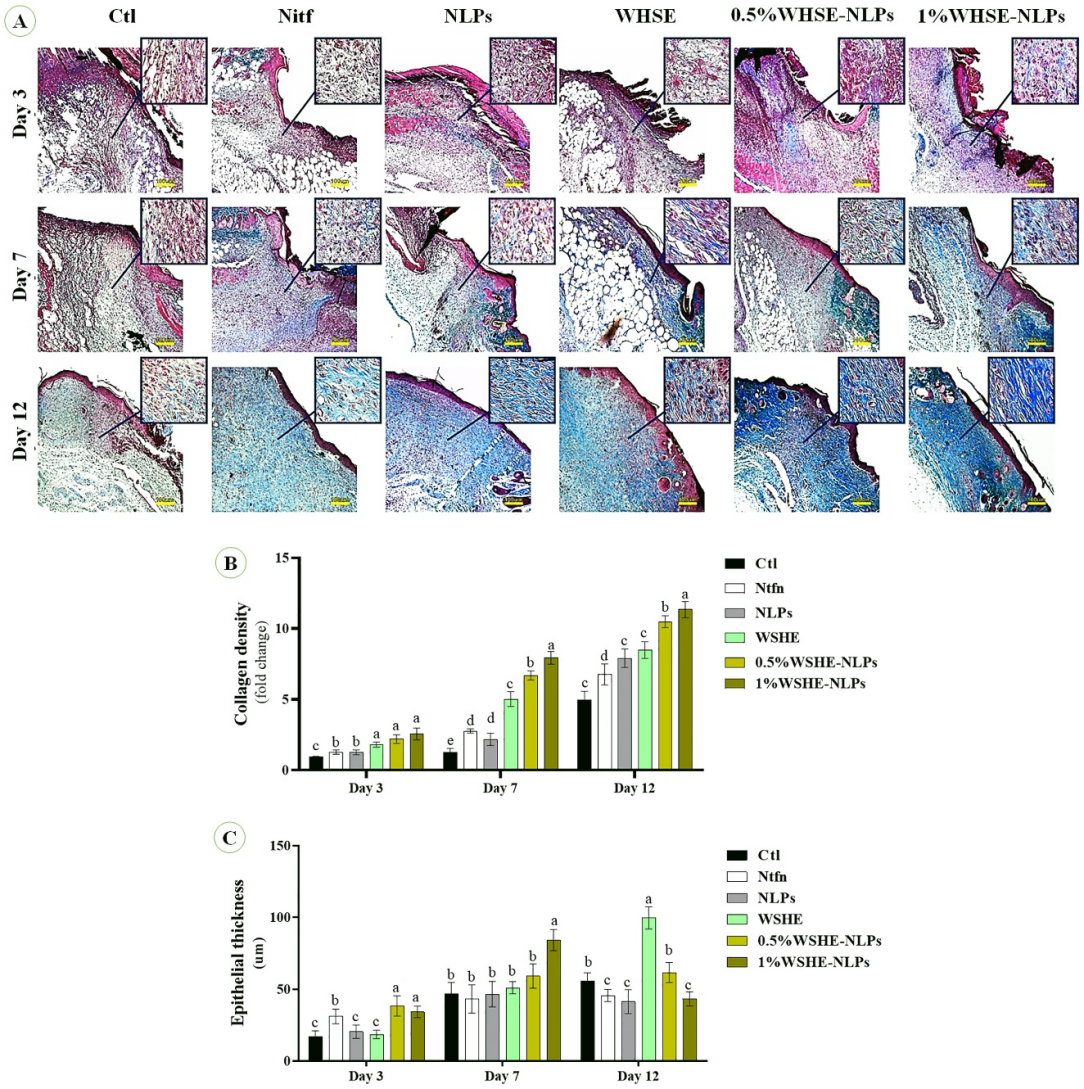
Representative photomicrographs on sampling days stained with the Masson’s trichrome staining. See the collagen bio-synthesis and well-recovered epidermis and dermis of the wound in treated groups

 According to Figure 6, mice treated with 1% WSHE-NLPs, 0.5% WSHE-NLPs, and WHSE had significantly higher collagen density compared to other groups. Additionally, collagen deposition was notably higher in the Ntfn group throughout the study when compared to the control group. On days 3 and 7, there was a significant increase in epithelial thickness in the 1% WSHE-NLPs, 0.5% WSHE-NLPs, and WHSE groups compared to the other groups. However, a decrease in epithelial thickness was observed in the 1% WSHE-NLPs and 0.5% WSHE-NLPs groups. These findings suggest that the treatments promote collagen production and accumulation, which aids in wound healing. The increase in collagen density and epithelial thickness facilitates the wound-healing process, with collagen contraction helping to reduce the wound area. The decrease in epithelial thickness on day 12 indicates improved initial epithelization.

###  The expression of CD31 and bFGF antibodies

**Figure 7:**
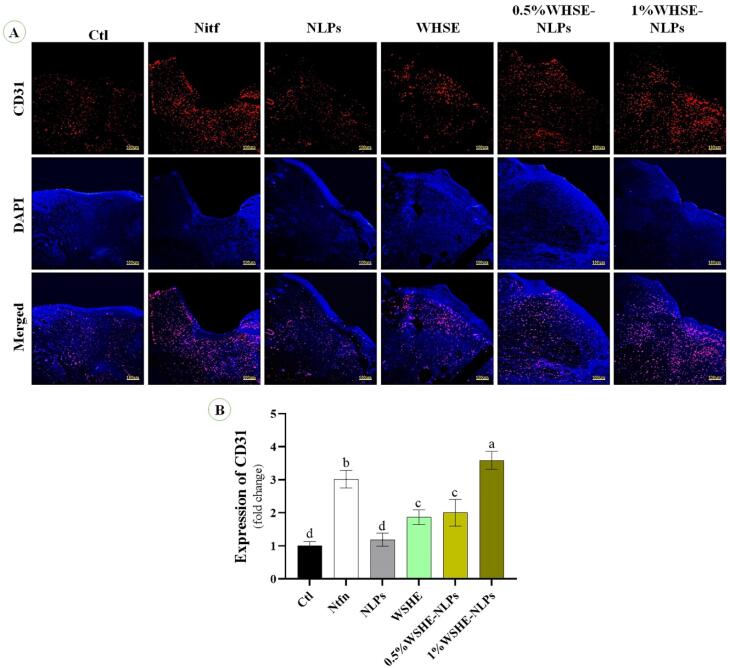
The effects of extract and its NLPs on the expression of CD31 (positive red area) and cell nuclei (blue). The quantitative analysis is based on CD31 staining. Different superscripts (a-e) show significant differences on the same day

**Figure 8:**
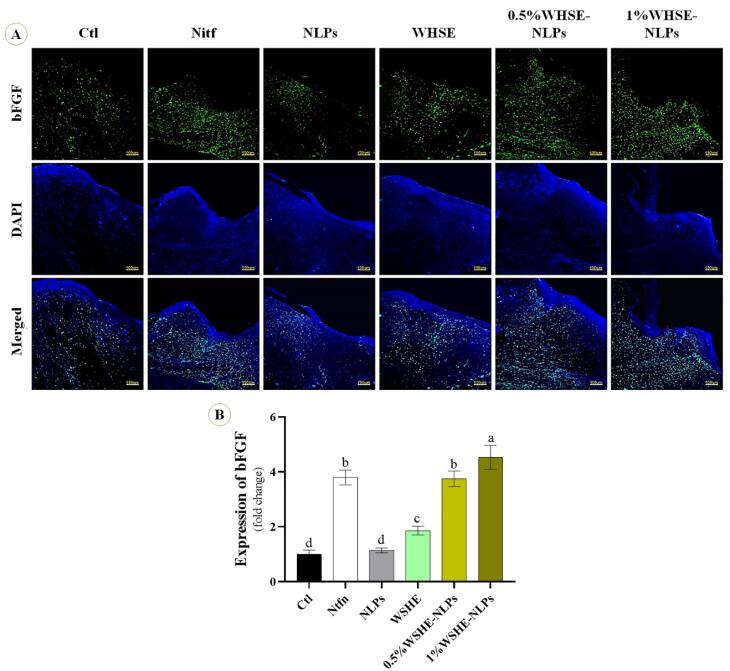
The effects of extract and its NLPs on the expression of bFGF (positive green area) and cell nuclei (blue); (b) The quantitative analysis based on bFGF staining. Different superscripts (a-e) show significant differences on the same day

 We observe the effect of the extract and its NLPs on CD31 and bFGF expression (Figures 7 and 8). Our results indicate that the levels of CD31 and bFGF were significantly higher (*P* < 0.05) in Ntfn and 1% WSHE-NLPs compared to other groups. Additionally, the expressions of bFGF and CD31 were higher in 0.5% WSHE-NLPs, WHSE, and NLPs than in control groups. bFGF is a mitogen factor that plays an important role in cell differentiation, proliferation, tissue regeneration, and chemotaxis during wound healing.^43^ Therefore, bFGF expedites the wound healing process, and the extract and its NLPs accelerate wound healing by increasing the expression of bFGF. CD31 plays a role in angiogenesis, which is enhanced with increased extract concentration.^44^ The rise in angiogenesis provides nutrients for the wound-healing process and accelerates the healing process. Loading WHSE into NLPs accelerates the wound healing process compared to the lack of coating status.

## Conclusion

 The study used a mouse model to test the effects of WHSE extract loaded into NLPs on wound healing and antibacterial properties. In vitro, findings showed that WHSE and its NLPs had antibacterial and antioxidant properties. In vivo, results indicated that ointments containing 1.00% of WHSE-NLPs significantly accelerated wound healing and enhanced collagen deposition and epithelialization more effectively than those containing 0.50%. Furthermore, the application of WHSE and its NLPs promoted faster wound healing by increasing the expression of CD31 and bFGF, as well as improving collagen deposition and epithelialization.

 Loading WHSE into NLPs made the wound healing process more efficient than the non-loaded form. Moreover, the pronounced effectiveness of WHSE-NLPS can be attributed to two main factors: (1) the protective role of the liposomal bilayer surrounding WHSE, which shields it from external conditions, thereby enhancing its impact on targeted tissues; and (2) the nanoscale size of NLPs, which allows for improved penetration through the stratum corneum, enabling access to the epidermis

 It’s important to note that the study was conducted on mice, so the results cannot be directly applied to humans. Before using these findings in human treatments, clinical studies are needed to validate their safety and effectiveness.

## Competing Interests

 The authors declare that they have no known competing financial interests or personal relationships that could have appeared to influence the work reported in this paper.

## Ethical Approval

 Ethics approval and consent to participate: The project was found to be in accordance with the ethical principles and the national norms and standards for conducting Medical Research in Iran, with Approval ID: IR.ZAUMS.REC.1397.313

## References

[1] Qu G, Xia T, Zhou W, Zhang X, Zhang H, Hu L (2020). Property–activity relationship of black phosphorus at the nano–bio interface: from molecules to organisms. Chem Rev.

[2] Rousselle P, Braye F, Dayan G (2019). Re-epithelialization of adult skin wounds: cellular mechanisms and therapeutic strategies. Adv Drug Deliv Rev.

[3] Liang Y, Chen B, Li M, He J, Yin Z, Guo B (2020). Injectable antimicrobial conductive hydrogels for wound disinfection and infectious wound healing. Biomacromolecules.

[4] Younis NS, Mohamed ME, El Semary NA (2022). Green synthesis of silver nanoparticles by the cyanobacteria Synechocystis sp: characterization, antimicrobial and diabetic wound-healing actions. Mar Drugs.

[5] Gaspar-Pintiliescu A, Stanciuc AM, Craciunescu O (2019). Natural composite dressings based on collagen, gelatin and plant bioactive compounds for wound healing: a review. Int J Biol Macromol.

[6] Saleem S, Muhammad G, Hussain MA, Altaf M, Bukhari SN (2020). Withaniasomnifera L: insights into the phytochemical profile, therapeutic potential, clinical trials, and future prospective. Iran J Basic Med Sci.

[7] Sharifi-Rad J, Quispe C, Ayatollahi SA, Kobarfard F, Staniak M, Stępień A (2021). Chemical composition, biological activity, and health-promoting effects of Withaniasomnifera for pharma-food industry applications. J Food Qual.

[8] Pavan Kumar Achar GS, Prabhakar BT, Rao S, George T, Abraham S, Sequeira N, et al. Scientific validation of the usefulness of Withaniasomnifera Dunal in the prevention and treatment of cancer. In: Akhtar MS, Swamy MK, eds. Anticancer plants: Properties and Application. Vol 1. Singapore: Springer; 2018. p. 285-301. 10.1007/978-981-10-8548-2_12.

[9] Paul S, Chakraborty S, Anand U, Dey S, Nandy S, Ghorai M (2021). Withaniasomnifera (L) Dunal (Ashwagandha): a comprehensive review on ethnopharmacology, pharmacotherapeutics, biomedicinal and toxicological aspects. Biomed Pharmacother.

[10] Alawdi SH, Shehab M, Al-Mekhlafi AG (2019). Formulation of herbal gel preparations from medicinal plants and evaluation of their wound healing activities. Saudi J Biomed Res.

[11] Lucia A, Guzmán E (2021). Emulsions containing essential oils, their components or volatile semiochemicals as promising tools for insect pest and pathogen management. Adv Colloid Interface Sci.

[12] Bilia AR, Isacchi B, Righeschi C, Guccione C, Bergonzi MC (2014). Flavonoids loaded in nanocarriers: an opportunity to increase oral bioavailability and bioefficacy. Food Nutr Sci.

[13] Gondim BL, Oshiro-Júnior JA, Fernanandes FH, Nóbrega FP, Castellano LR, Medeiros AC (2019). Plant extracts loaded in nanostructured drug delivery systems for treating parasitic and antimicrobial diseases. Curr Pharm Des.

[14] Savaghebi D, Barzegar M, Mozafari MR (2020). Manufacturing of nanoliposomal extract from Sargassum boveanum algae and investigating its release behavior and antioxidant activity. Food Sci Nutr.

[15] Akhlaghi M, Eftekharivash L, Taebpour M, Afereydoon S, Ebrahimpour M, Zarezadeh Mehrizi M (2022). Improving the therapeutic performance of glycyrrhiza glabra hydroalcoholic extract using liposomal nano-carriers and their characterization. Dis Diagn.

[16] Gupta PK, Priyanka V, Hiremath L, Kumar SN, Srivastava AK (2018). Extraction of phytochemicals from Eucalyptus spp & Withaniasomnifera and their biological testing. Am J Microbiol Res.

[17] Hou M, Hu W, Xiu Z, Shi Y, Hao K, Cao D (2020). Efficient enrichment of total flavonoids from Pteris ensiformis Burm extracts by macroporous adsorption resins and in vitro evaluation of antioxidant and antiproliferative activities. J Chromatogr B Analyt Technol Biomed Life Sci.

[18] Shahriar M, Hossain MI, Sharmin FA, Akhter S, Haque MA, Bhuiyan MA (2013). In vitro antioxidant and free radical scavenging activity of Withaniasomnifera root. IOSR J Pharm.

[19] Zeinali M, Abbaspour-Ravasjani S, Soltanfam T, Paiva-Santos AC, Babaei H, Veiga F (2021). Prevention of UV-induced skin cancer in mice by gamma oryzanol-loaded nanoethosomes. Life Sci.

[20] Abedi Gaballu F, Abbaspour-Ravasjani S, Mansoori B, Yekta R, Hamishehkar H, Mohammadi A (2019). Comparative of in-vitro evaluation between erlotinib loaded nanostructured lipid carriers and liposomes against A549 lung cancer cell line. Iran J Pharm Res.

[21] Mohammadi M, Ghanbarzadeh B, Hamishehkar H (2014). Formulation of nanoliposomal vitamin D3 for potential application in beverage fortification. Adv Pharm Bull.

[22] Farahpour MR, Mirzakhani N, Doostmohammadi J, Ebrahimzadeh M (2015). Hydroethanolic Pistacia atlantica hulls extract improved wound healing process; evidence for mast cells infiltration, angiogenesis and RNA stability. Int J Surg.

[23] Farahpour MR, Hesaraki S, Faraji D, Zeinalpour R, Aghaei M (2017). Hydroethanolic Allium sativum extract accelerates excision wound healing: evidence for roles of mast-cell infiltration and intracytoplasmic carbohydrate ratio. Braz J Pharm Sci.

[24] Farahpour MR, Sheikh S, Kafshdooz E, Sonboli A (2021). Accelerative effect of topical Zataria multiflora essential oil against infected wound model by modulating inflammation, angiogenesis, and collagen biosynthesis. Pharm Biol.

[25] Farahpour MR, Fathollahpour S (2015). Topical co-administration of flaxseed and pistachio ointment promoted wound healing; evidence for histopathological features. Comp Clin Pathol.

[26] Liao F, Chen L, Luo P, Jiang Z, Chen Z, Wang Z (2020). PC4 serves as a negative regulator of skin wound healing in mice. Burns Trauma.

[27] Were LM, Bruce B, Davidson PM, Weiss J (2004). Encapsulation of nisin and lysozyme in liposomes enhances efficacy against Listeria monocytogenes. J Food Prot.

[28] Gibis M, Ruedt C, Weiss J (2016). In vitro release of grape-seed polyphenols encapsulated from uncoated and chitosan-coated liposomes. Food Res Int.

[29] Geethalakshmi R, Sakravarthi C, Kritika T, Arul Kirubakaran M, Sarada DV (2013). Evaluation of antioxidant and wound healing potentials of Sphaeranthusamaranthoides Burmf. Biomed Res Int.

[30] Tsuchiya H, Sato M, Miyazaki T, Fujiwara S, Tanigaki S, Ohyama M (1996). Comparative study on the antibacterial activity of phytochemical flavanones against methicillin-resistant Staphylococcus aureus. J Ethnopharmacol.

[31] Martin A (1996). The use of antioxidants in healing. Dermatol Surg.

[32] Bates SH, Jones RB, Bailey CJ (2000). Insulin-like effect of pinitol. Br J Pharmacol.

[33] Bhatnagar M, Sisodia SS, Bhatnagar R (2005). Antiulcer and antioxidant activity of Asparagus racemosus Willd and Withaniasomnifera Dunal in rats. Ann N Y Acad Sci.

[34] Pal A, Naika M, Khanum F, Bawa AS (2012). In-vitro studies on the antioxidant assay profiling of root of Withaniasomnifera L(Ashwagandha) Dunal: part 2. Agric Conspec Sci.

[35] Ly HT, Pham Nguyen MT, Nguyen TK, Bui TP, Ke X, Le VM (2020). Phytochemical analysis and wound-healing activity of noni (Morinda citrifolia) Leaf Extract. J Herbs Spices Med Plants.

[36] Bisht P, Rawat V (2014). Antibacterial activity of Withaniasomnifera against gram-positive isolates from pus samples. Ayu.

[37] Kumari M, Gupta RP (2015). In vitro antibacterial effect of Withaniasomnifera root extract on Escherichia coli. Vet World.

[38] Gortzi O, Lalas S, Chinou I, Tsaknis J (2008). Reevaluation of bioactivity and antioxidant activity of Myrtus communis extract before and after encapsulation in liposomes. Eur Food Res Technol.

[39] Al-Henhena N, Mahmood AA, Al-Magrami A, Nor Syuhada AB, Zahra AA, Summaya MD (2011). Histological study of wound healing potential by ethanol leaf extract of Strobilanthes crispus in rats. J Med Plants Res.

[40] Phillips GD, Whitehead RA, Knighton DR (1991). Initiation and pattern of angiogenesis in wound healing in the rat. Am J Anat.

[41] Muhammad AA, Pauzi NA, Arulselvan P, Abas F, Fakurazi S (2013). In vitro wound healing potential and identification of bioactive compounds from Moringa oleifera Lam. Biomed Res Int.

[42] Juneja K, Mishra R, Chauhan S, Gupta S, Roy P, Sircar D (2020). Metabolite profiling and wound-healing activity of Boerhaviadiffusa leaf extracts using in vitro and in vivo models. J Tradit Complement Med.

[43] Ly HT, Pham Nguyen MT, Nguyen TK, Bui TP, Ke X, Le VM (2020). Phytochemical analysis and wound-healing activity of noni (Morindacitrifolia) Leaf Extract. J Herbs Spices Med Plants.

[44] Kobayashi F, Matsuzaka K, Inoue T (2016). The effect of basic fibroblast growth factor on regeneration in a surgical wound model of rat submandibular glands. Int J Oral Sci.

[45] Karnam K, Sedmaki K, Sharma P, Routholla G, Goli S, Ghosh B (2020). HDAC6 inhibitor accelerates wound healing by inhibiting tubulin mediated IL-1β secretion in diabetic mice. BiochimBiophys Acta Mol Basis Dis.

